# Accurate genome-wide phasing from IBD data

**DOI:** 10.1186/s12859-022-05066-2

**Published:** 2022-11-23

**Authors:** Keith Noto, Luong Ruiz

**Affiliations:** Ancestry DNA, San Francisco, USA

**Keywords:** Genotype phasing, Long-range phasing, IBD

## Abstract

As genotype databases increase in size, so too do the number of detectable segments of *identity by descent* (IBD): segments of the genome where two individuals share an identical copy of one of their two parental haplotypes, due to shared ancestry. We show that given a large enough genotype database, these segments of IBD collectively overlap entire chromosomes, including instances of IBD that span multiple chromosomes, and can be used to accurately separate the alleles inherited from each parent across the entire genome. The resulting phase is not an improvement over state-of-the-art *local* phasing methods, but provides accurate long-range phasing that indicates which of two haplotypes in different regions of the genome, including different chromosomes, was inherited from the same parent. We are able to separate the DNA inherited from each parent completely, across the entire genome, with 98% median accuracy in a test set of 30,000 individuals. We estimate the IBD data requirements for accurate genome-wide phasing, and we propose a method for estimating confidence in the resulting phase. We show that our methods do not require the genotypes of close family, and that they are robust to genotype errors and missing data. In fact, our method can impute missing data accurately and correct genotype errors.

## Background

*Phasing* refers to the separation of maternally and paternally inherited DNA. Genotype data are most often generated in an unphased state, because genotyping technologies work at a local level, determining the diploid genotype of one single-nucleotide polymorphism (SNP) at a time. However, phased data are often significantly more valuable. For example, some genotype–phenotype relationships depend on how certain variants are situated across both copies of a homologous genomic region, and phase information is beneficial in the study of genomic diversity and for the purpose of haplotype matching [1]. In applications where pedigrees are available, there is an advantage in knowing which genomic variants of interest are inherited from the same parent by having genomic data phased across the whole genome, and in any application regarding the ancestry of an individual it is advantageous to consider the entire genome, and to consider the DNA inherited from each parent as having its own ancestry.

The advantages of phased data are known and there are several methods of modeling and analyzing genotype data for the purpose of phasing, e.g., [2–6] but they are often only able to phase well at a local level–the two parental copies are inevitably swapped many times across the genome.

A simple and common way to separate the haploid DNA inherited from each parent is to compare them to the data of the parents themselves. This is *duo*-phasing, or *trio*-phasing if both parents’ genotype data are available. However, the parents’ data may be expensive or impossible to obtain, and if parental data are missing or all three individuals are heterozygous at a site, the phase cannot be resolved with Mendelian logic alone, and one must defer to a model.

**Fig. 1:**
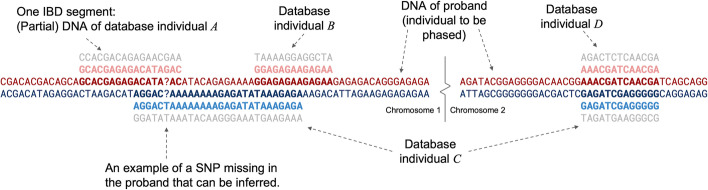
An illustration of the genotype data used for phasing. The DNA of the proband consists of two haploid genotypes across all chromosomes. IBD segments also consist of two haplotypes, one of which is identical to one of the proband’s haplotypes. Note that each IBD segment (partial diploid) is consistent with exactly one of the proband’s haplotypes, and that we can infer that the same haplotype in the proband is shared with individual *A* and individual *B*, even though those segments do not overlap (because they both overlap with individual *C*). Individual *C*’s two IBD segments are on different chromosomes and it is more likely than not that the haplotypes shared with individual *C* are inherited from the same parent (with dozens or hundreds of multi-chromosome IBD segments, inter-chromosome phasing becomes clearer)

*Identity by descent* (IBD) occurs when one of a person’s two haplotypes is identical to one of another person’s in a segment of the genome because the two share a common ancestor. It has been previously shown that IBD data can be used to phase and determine the parent from which haplotypes are inherited [7]. The approach is essentially to identify segments of IBD and use them as “surragate parents,” duo- or trio-phasing those parts. This is the crux of our approach as well, except that in our scenario, there are enough IBD segments that we can expect most IBD segments to overlap others on both sides of the family, and most sites to overlap multiple IBD segments, each providing information on which allele is part of a shared haplotype even if those IBD segments are between unphased diploids. Figure 1 shows a small illustration of the type of data we use.

When IBD segments overlap, they form a collective block of the genotype for which there is essentially only one way to assign each IBD segment to one parental haplotype or the other, and infer the phase of the proband. Given enough overlapping IBD data, these blocks extend to the full size of a chromosome, although they are not guaranteed to do so. We refer to these blocks as *subclusters*: a block of overlapping IBD segments separated into two parental groups.

The task that remains is to determine which parental group of each subcluster corresponds to the same parent in the other subclusters. That is, we must phase the subclusters into larger *superclusters*, and align the parental groups of each subcluster. Note that we cannot determine from autosomal DNA which parent is the mother. Our goal is to combine all subclusters into one supercluster that separates all the IBD segments into two parental groups, *A* and *B*, without necessarily identifying the parents. We illustrate this task in Additional file 1: Figure S1.

The primary mechanism for aligning the parental groups of subclusters on different chromosomes is based on individuals that share IBD segments across multiple subclusters. For example, in Fig. 1, individual *C* shares DNA with the proband on chromosomes 1 and 2. It is generally more likely that both the proband and individual *C* inherited both of the shared haplotypes from the same side of the family rather than (e.g.) individual *C* sharing DNA with the proband’s mother on chromosome 1 and with the proband’s father on chromosome 2, although the latter case is possible. However, when our IBD data consist of many instances where the proband shares DNA across multiple subclusters, we can phase the subclusters correctly by maximizing the number of instances where multi-segment IBD data are inherited from the same parent in a supercluster.

Depending on the amount and distribution of IBD data, it is possible that some parts of the genome do not overlap any IBD data and cannot be phased (in which case we may default to the phase inferred by models), or that some subclusters cannot be connected to others through multi-segment IBD (in which case we will have multiple superclusters of varying size), but we evaluate our method on the genome-wide phase that results in all scenarios and characterize the IBD data required to apply our approach. We use this method to phase 30,000 child-father-mother trios using IBD segments detected among a database of 12,755,111, excluding IBD shared with parents. We call our proposed method **IBDphase**. In the Sections that follow, we show the phase accuracy of the method and that this approach can be used to effectively impute missing data and correct genotype errors in data. We provide details of our approach, including how we make our methods robust to potential genotype errors in the proband genotypes, and propose a method for estimating the accuracy of the resulting phase.

## Results

The primary methodology we use to evaluate our approach is to use the genotype data of 30,000 child-parent-parent trios, identify IBD shared between the children in this test set and a database of over 12 million genotypes (for this we use unphased data [8]), apply IBDphase without using the parents’ genotype or IBD data, and then compare the resulting phase to trio phase using the parents’ data. The statistic we are most interested in is the global separation of the alleles inherited from each parent: when we phase the genome into two haploid sequences, to what extent did we correctly separate all of the alleles inherited from mother from all the alleles inherited from father? We also measure local phase accuracy, and the accuracy of the assignment of IBD segments to parental sides. We describe our experimental methodology in detail in the Experimental Methodology Section below.

We pre-phase our test set using Eagle v2.4.1 [6] (using only the cohort of 30,000 individuals). We do not consider the resulting phase accuracy to be the state-of-the-art standard for comparison, but pre-phasing our data is a necessary step for our approach because it provides a default to make phasing decisions at sites where there are no overlapping IBD segments. Indeed, methods like Eagle are not suited to separate the DNA inherited from each parent at a genome-wide level. In contrast, IBDphase is able to separate the DNA inherited from each parent in our test set with an average accuracy over 95% (and a median accuracy of nearly 98%). Phase accuracy results are shown in Table 1 (see also Additional file 1: Table S5 and Figures S6 and S7 for complete statistics). In terms of phase accuracy at the local level, the phase produced by IBDphase in these experiments has a median switch error rate of 0.26%, and most of the test set have at least 99% of their heterozygous SNPs phased in complete agreement with trio phase in a region that measures at least one centimorgan (this is a measure of local phase accuracy that does not penalize for small areas of the genome that are phased very poorly). However, we find that state-of-the-art methods [4, 6, 9] outperform local IBDphase if the cohort size is large enough (see Additional file 1: Table S13).

IBDphase also labels each IBD segment as being on one side of the family or the other. When we compare the groups created to the IBD segments we identify using the parents’ DNA, we find that the average IBD segment assignment error is 3.4% and the median error is 0.67%. This measure of error is comparable and correlated with genome-wide phasing error (See Additional file 1: Figure S13), but does not penalize for portions of the genome that do not overlap IBD segments.

**Table 1:** Phase Accuracy. The genome-wide phase accuracy of IBDphase. Accuracy is measured using trio phase as the standard of true phase, using only SNPs where the phase can be unambiguously inferred from the trio (i.e., where at least one parent is homozygous). Global error is the rate at which the phase differs from trio phase (keeping only one haplotype assigned to one parent across the genome, but assuming the more favorable haplotype of two choices). Switch error rate is the frequency with which the phase of a heterozygous SNP differs from that of the previous heterozygous SNP with respect to trio phase. The third accuracy measure is the proportion of SNPs that belong to segments where there are no phase switches for at least 1 centimorgan. Some measures depend on SNP density, and we consider 416,176 SNPs across the autosome. The median global phase error of the pre-phased data is 48%, and the median phase switch rate and median proportion of SNPs in 1 cM+ runs is 1.08% and 95% respectively

Criteria	IBDphase median (%)	IBDphase mean (%)
Global phase error	2.09	4.93
Phase switch error rate	0.26	0.33
Proportion of SNPs in 1 cM+ runs	99.01	98.64

### Estimating the accuracy of resulting phase

IBDphase performs better when the genomic database is large, when many IBD segments are discovered in it, when a large proportion of sites overlap at least a few IBD segments, and when there are close genetic relationships to provide long IBD segments and help phase across multiple chromosomes. Table 2 shows that all these measures are strongly correlated, and provides an estimate of how performance on this database may continue to improve. The overall genome-wide phase error is high when the database is too small (see Additional file 1: Table S5 and Figures S4, S6, S7, and S8 for more information, and Additional file 2 for detailed data) and global error rate at sites that do not overlap any IBD is high, as can be expected (the median global error rate among those sites is 30.5%).

**Table 2:** Performance and IBD statistics as database size increases. As the size of the genomic database in which IBD is identified increases, so does the global phase accuracy, number of close genetic relationships, proportion of the genome that overlaps at least a few IBD segments, and the overall number of IBD relationships

Database size (Millions)	Median phase error (%)	Median number of 400 + cM relations	Median proportion of SNPs that overlap at least 5 IBD segments (%)	Median number of IBD relations
1.00	36.8	1 (mean 1.32)	67.7	5024
2.00	22.1	1 (mean 1.58)	82.7	10,003
5.00	6.99	2 (mean 2.43)	94.9	24,623
12.76	2.09	3 (mean 4.31)	98.7	60,778

The correlation between these statistics and observed phase accuracy at the level of an individual proband is much weaker, however (e.g., see Additional file 1: Figure S8), but we can combine features of the phasing process to train a predictor that can help estimate the confidence we have in the phase quality of any individual proband (see the “Methods” section for details). Table 3 shows the most informative features. The Pearson correlation between the prediction from our error model on this test set and the observed global phase error is 0.66 and the Spearman correlation is 0.45.

There clearly is a relationship between the number of close IBD relationships and the performance of IBDphase, however we note that IBDphase performs well without close relationships. 42% of our test set have no IBD relationships that share more than 1000 centimorgans, and the median genome-wide phase error among those is 3.6%. 11% of our test set have no IBD relationships of more than 400 cM, and the median error rate among them is 6.8%. Furthermore, the number of close relationships is less correlated with global error than our predictor (e.g., the Pearson correlation between the number of 400 cM relationsips and global accuracy is 0.31, and it is 0.25 between the number of 1000 cM relationships and accuracy).

**Table 3:** Features used to estimate phase confidence. The top 10 features, ranked by their importance (GINI importance, as calculated by *scikit-learn* [10] for random forests)

Feature	Importance
Proportion of database individuals with IBD segments assigned to both sides of the family, both on the largest supercluster	0.388
Proportion of IBD segments that are partially assigned to one parental side and partially to the other	0.108
Number of close family members that do not share DNA with all other close family	0.103
(log) number of database individuals with shared DNA	0.088
(log) number of IBD segments assigned to the largest supercluster	0.081
Number of missing edges in close family network (pairs of close family that do not share IBD with each other)	0.032
Proportion of the genome overlapped by at least one IBD segment	0.030
Ratio of the number of database individuals with shared DNA on one IBD segment to the number with shared DNA on multiple IBD segments	0.029
Proportion of the genome overlapped by at least two IBD segments	0.021
Proportion of IBD segments assigned to the largest supercluster	0.018

The number of available IBD relationships in our database depends on how much or how little IBD is allowed by the IBD detection process used. In our experiments, we discard any IBD relationships shorter than 8 centimorgans. If we allow shorter IBD relationships, we risk losing accuracy [8], and indeed the performance of IBDphase is slightly worse if we allow 5 or 6 cM relationships However, the optimal threshold may be closer to 10 cM. (See results in Additional file 1: Table S14.)

A large proportion of our database consists of individuals with European ancestry and as expected, the performance of IBDphase is worse on individuals whose ancestry is underrepresented in our database. There is strong evidence that the decreased performance in such individuals is due to the fact that there is a smaller number of IBD segments associated with them. For example, if we classify each test set individual according to the major geographic region that explains the largest portion of their DNA according to AncestryDNA estimates (there are 12 such regions with at least 20 representative individuals, see Additional file 1: Table S10 and Additional file 2), we observe that the proportion of the genome that is overlapped by at least five IBD segments is a much stronger predictor of phase accuracy than the population designation. Specifically, if we divide the amount of genome coverage into 12 exclusive ranges, the mutual information between genome coverage and the decile of the phase error is 0.24, whereas the mutual information between the population designation and the phase error is 0.027. Indeed, if we restrict our evaluation to individuals with a minimum genome coverage, the performance improves across all groups (Additional file 1: Table S10). We also observe that the performance of IBDphase in admixed individuals such as African Americans (in this case, defined by having at least 20% African and 20% European estimated assignment and at least 80% combined African and European assignment) is comparable to the rest of the test set (median global phase error 2.60%, n=1587), and the median global phase error in Latinx test set individuals (10% European, 10% from the Americas, 50% combined European and American) is 2.09% (n=1039). These observations suggest that as the database continues to grow, the performance of IBDphase increases, regardless of demography.

### Imputation and correction of genotype errors

IBDphase can also improve imputation accuracy and correct genotype errors because it observes the genotypes of several individuals at the same site (the proband and overlapping IBD segments). In cases where overlapping IBD segments imply a different genotype than the one in the input or imputed in the pre-phased data, IBDphase will override the call. We test the effectiveness of these corrections by artificially altering some genotypes and setting some genotypes to missing before performing the same phasing experiment as above on 5000 of our original 30,000-individual test set, selected uniformally at random. In these experiments, we evaluate IBDphase by whether or not it is able to replace the perturbed genotypes with the original. Since artificial genotype errors affect whether or not IBD is detected, we evaluate only on SNPs that are not used in IBD detection (although results are similar when we evaluate on all SNPs–see Additional file 2).

In the scenario where we set a genotype to missing, a genotype is imputed during the pre-phasing process (again, we use Eagle [6], this time on the 5000 individuals whose data we perturbed and the 25,000 unaltered members of the original test set). When there are not enough overlapping IBD segments on either side of the family, IBDphase always keeps that imputed call, which is correct 97% of the time. When there is enough overlapping IBD on one side only, IBDphase changes the imputed call 1% of the time, and the change is correct 97% of the time. When there is enough IBD on both sides, IBDphase changes the call 0.5% of the time, and the change is correct 95% of the time. In this experiment, IBDphase reduced the number of imputation errors from 86,935 to 40,689 (a 53% reduction). Note that when there is evidence on only one side, IBDphase only overrides homozygous calls, but when there is evidence on both sides, IBDphase can make a stronger change, albeit with slightly less accuracy. The final IBDphase call is correct 97% of the time when there is no IBD evidence on either side, 99.1% of the time when there is evidence on one side, and 99.8% of the time when there is evidence on both sides.

In the scenario where we replace a genotype with a (presumed) genotype error, the pre-phased data always reflects the perturbed genotype, and if there is not enough overlapping IBD on either side of the family, IBDphase keeps that call, which is always incorrect in this simulation. However, when there is enough overlapping IBD on one side of the family, IBDphase overrides the call 50% of the time, and the change is correct 100% of the time (with IBD on one side, IBDphase only overrides homozygous calls). When there is enough overlapping IBD on both sides, IBDphase overrides the call 79% of the time and is correct 99.993% of those. IBDphase corrected 65% of all genotype errors in total. When the given genotype was not altered for this experiment, IBDphase overrode the call in 0.017% of the instances when there was enough overlapping IBD on one side, and 0.020% of the instances when there was enough IBD on both sides. Accounting for all SNPs in this experiment, perturbed or not, IBDphase reduced the number of genotype errors by 55%. Note that our “ground truth” for measuring genotype errors is the original genotype calls before perturbation. We note that if those calls are in error (in reality, but unknown to us), we do measure them as false positives, which may make our accuracy slightly underestimated.

## Discussion

Our approach is comparable to the techniques used in Kong et al. [7] but our aim is to separate maternally and paternally inherited DNA across the entire genome without requiring an elevated level of IBD sharing in any specific population, as the deCODE [7] study has in Iceland. IBDphase does not use full siblings at all in its analysis, as they share DNA on both sides of the family, but multiple siblings used at once could identify breakpoints between DNA inherited from different grandparents, as shown in the deCODE study [7], but we have not experimented with the use of multiple siblings and focus instead on the generality of our approach that does not require the genotypes of any close family. After IBD segments are separated into parental groups, breakpoints become much more apparent, where numerous IBD segments end and another set of IBD segments begin, which are likely to be places where a recombination event occurred in one of the proband’s ancestors. One area for further study is the extent to which these groups can be associated with specific ancestors and how large a database is necessary.

One of the potential pitfalls with our method is an instance where a significant number of individuals share DNA with both of the proband’s parents. These are often cases where the proband’s parents are not closely related, but do have ancestry that is similar enough that they share DNA with the same individuals due to identity by state or founder effects, or from influences of SNP density and population-specific allele frequencies. Such scenarios add noise to the information IBDphase uses to phase subclusters, as evidenced by the facts that (i) discarding some of the shortest IBD relationships (e.g., 8–10 cM) may be beneficial (see Additional file 1: Table S14), and (ii) the most informative feature in estimating the phase quality is the number of database individuals that have multiple segments which IBDphase is forced to put on opposite sides of the family (Table 3). Distinguishing IBD segments that are not informative in this way from those that are informative is a difficult open problem and effective solutions are likely to provide the largest benefit to the performance of IBDphase.

## Conclusion

IBDphase provides excellent genome-wide phasing, as well as the assignment of IBD segments to parental haplotypes, provided a large enough IBD database. Our method is able to phase, impute, and correct errors with high accuracy at a local genomic level, and accurately identify the corresponding parents in phased data across chromosomes, resulting in complete separation across the genome of the DNA inherited from each parent, with an accuracy usually above 95% (accuracy is over 95% more than 75% of the time, and above 99% over 30% of the time). IBDphase also labels IBD segments in a cluster hierarchy that indicates the genomic locations of the most confident resulting phase, and provides an error model that can predict with reasonable accuracy whether or not the phase is likely to be reliable genome-wide.

## Methods

### Our IBD-phase approach

Our approach has the following steps (for each proband we wish to phase):

1. Load the IBD coordinates (specifying which individuals in the database share IBD with the proband), as well as the genotype data for those individuals at those coordinates, all genotype data for the proband, and all genotype data for close family of the proband (if any). If available, load phased data for the proband (these data will provide the “default” phase for sites that cannot be resolved from IBD segments. If not available, the default phase is arbitrary).
2. Compare the genotypes of close family to each other and determine if they are potentially other descendants of the proband’s parents (we will discard those).
3. Separate the IBD segments on each chromosome into two parental groups, potentially breaking some into smaller pieces where data are inconsistent.
4. Identify subclusters: groups of IBD segments such that each significantly overlaps with others in the same group.
5. Phase the subclusters and group them into superclusters and determine which parental group of each subcluster corresponds to which parental group of the supercluster.
6. Phase the proband genotype across the genome using the genotype information in the separated parental groups.

We describe each of these steps in turn, and they are also illustrated in the pseudocode appendix in Additional file 1. First, we load data. For efficiency, it is essential that genotype data are stored in binary format, indexed by individual, and all use the same coordinates (i.e., set of SNPs) as the IBD data. Then, for all IBD segments, we can seek and read only the segments of genotype data that correspond to the IBD segments.

Second, we compare the genotypes of (relatively) close relationships. We consider a close relation to be anyone who shares a significant amount of IBD (we use a 400 centimorgan threshold) so that we can safely assume that if they are related to both of the proband’s parents, we will detect at least some (at least 8 cM) of IBD with all of the other close relations.

Individuals who are descendants of the proband’s parents (e.g., a nephew) are unreliable data for phasing the proband, because they will share DNA with the proband throughout the genome on both sides of the family. We discard any IBD segments from close family that shares IBD with all other close family because they are potentially descendants of both the proband’s parents. We note that it is possible that we may discard IBD shared on only one side of the proband’s family. We also discard IBD segments shared with an identical twin or full sibling of the proband. These cases are identified by patterns of IBD2 throughout the genome. Specificially, we measure runs of consecutive IBD2 (i.e., the proband and the database individual have the same genotype for consecutive SNPs) that are at least 5 cM in length, we measure the overall rates of IBD1 (sharing at least one allele) and IBD2 (sharing both alleles) across the genome, as well as the 8 cM segments of any sharing (IBD1 or IBD2) that we identify for all database individuals. We find that, in terms of these measurements, the distinction between siblings, identical twins, and other close relatives is very distinct, with siblings usually sharing about 25% of DNA in runs of IBD2, identicial twins sharing virtually 100% of DNA in runs of IBD2 and other close relationships sharing very little IBD2. However, to account for underestimates due to very short runs or genotyping errors, we consider a database individual to be an identical twin of the proband if 90% of the genome consists of sharing in runs of IBD2. We consider a database individual to be a full sibling of the proband if the total amount of sharing across the genome (IBD1 or IBD2) is at least 1,300 cM, the amount of DNA shared in detected runs of IBD2 sums to at least 9% of the genome, but the overall rate of IBD1 across the genome is less than 99%. In contrast to descendants of the proband’s parents, close relations that are not discarded are particularly useful in phasing the proband because they will share a significant amount of DNA with the proband exclusively on one side of the family. Note that close family are not required for our approach.

The next step is to separate the IBD segments into two parental groups such that segments that overlap each other and share the opposite parental haplotype are separated into opposite groups. (an illustration of our approach is shown in Additional file 1: Figure S2). To make this efficient, we rely on the fact that (with very few exceptions which are discussed below) any two overlapping IBD segments will either share the (i) same or (ii) the opposite haplotype with the proband, and will therefore (i) always be homozygous for the same allele or (ii) always be homozygous for the opposite allele at sites where the proband is heterozygous and both IBD segments are homozygous. For example, in Fig. 1, in all the places where the proband is heterozygous but two IBD segments are both homozygous at the same site, the two IBD segments are homozygous for the opposite alleles, implying that they do not share the same haplotype. Our approach is to scan the proband’s heterozygous sites and divide the overlapping IBD segments into two groups: those that are homozygous for one allele, and those that are homozygous for the other. These represent the two sides of the proband’s family, and our aim is to make sure that all IBD segments are consistently assigned to the same group. In practice, there are conflicts to resolve which arise either from genotype errors, or from the aforementioned fact that several IBD segments are likely to extend beyond the genomic positions where they truly share a haplotype. We account for this by downweighting the information contribution of an IBD segment if a SNP site in question is near either end of the segment (see Additional file 1: Figure S11). At each site that is heterozygous in the proband, we choose to phase the site in whichever way maximizes the (weighted) number of IBD segments that will stay in the same parental group to which they were assigned at the previous heterozygous site in the proband. If there are any IBD segments that must change sides of the proband’s family as a result, we break that segment into two. In practice, few segments are significantly broken and we are left with an improved estimate of their positions (See Additional file 1: Table S3 for relevant statistics from our experiments). At sites where the phase cannot be determined by the parental group assignments, (e.g., if an overlapping IBD segment is not yet assigned to a group, and there are no assigned segments that are homozygous at the same site), we follow the phase described by the pre-phased data. This means that we place the alleles that are in the same haplotype in the pre-phased data on the same side of the family that they were placed in the previous heterozygous site in the proband. There is one potential pitfall that we must account for explicitly: if the proband is *erroneously* called heterozygous, then the procedure described above is likely to break up segments unnecessarily and assign them to the wrong parental groups. It is not always clear when examining individual SNPs that the proband is homozygous but called in error, and once the parental groups of IBD segments have become corrupted as a result, they can cause further errors downstream of the erroneous site. To address this issue, we employ a lookahead scheme. We associate a cost with breaking up an IBD segment (i.e., changing its assignment from one parental group to the other), and a cost with ignoring a site.

If a SNP that is called heterozygous in the proband requires that some shared IBD segments (we use a threshold of 1.0 segments after weighting, see Additional file 1: Figure S11) must change from one parental group to the other relative to the previous proband heterozygote, we examine the heterozygous sites closest to the SNP in question (we examine 20 proband heterozygous sites in either direction) to detect if their parental assignments to IBD segments significantly disagree with those of the site in question.

For instance, if an upstream proband heterozygous site divides overlapping IBD segments into two groups, some of which are homozygous for one allele, and some of which are homozygous for the other, and a downstream site separates IBD segments into those same two groups, yet the current site puts all IBD segments into one group, this is evidence that the current site in fact homozygous in the proband.

Whenever enough IBD segments must change parental groups based on their homozygosity at a site, IBDphase examines the next 20 sites that are heterozygous in the proband in the upstream direction and 20 sites in the downstream direction and counts the number of IBD segments that must change parental groups, each weighted in the same way as described above (Additional file 1: Figure S11). We examine multiple sites in this step because we only consider homozygous genotypes in the overlapping IBD segments. If the weighted sum of segments that change groups as a result of this procedure exceeds the cost of ignoring a site (we use a cost threshold of 1.0), then we effectively ignore the site, keep the same parental group assignments to the IBD segments as in the previous proband heterozygous site, and move to the next. In our experiments, we ignore about $0.3\%$ of SNPs.

Note that if an IBD segment shares *both* haplotypes (“IBD2”), it will not affect the phase. All sites that are heterozygous in the proband will be heterozygous in the other genome and the segment will not be assigned to either side of the family. Only in cases where an IBD2 segment has several genotyping errors and no other IBD1 segments to “outvote” it will IBD2 data infer incorrect phase. Full siblings are the only relationships with significant IBD2, and these are straightforward to identify and discard.

The next step in our approach is to delineate the IBD segments that make up subclusters. These are collections of IBD segments that overlap on the genome enough to determine whether they are on the same side of the family or the opposite. That determination is primarily based on homozygous sites in IBD segments because there is only one allele which therefore must be the allele shared with the proband. We cannot assume that our IBD data perfectly delimit where a shared haplotype begins and ends. Such IBD data are typically generated by comparing pairs of diploid genotypes in unphased or imperfectly phased data, and IBD is commonly estimated as extending beyond the genomic positions where the identity is due to a shared ancestor. We therefore insist that each IBD segment in a subcluster overlaps another in the same subcluster by a minimum number of sites (we use 10.0 sites, after weighting each using the same weighting scheme applied above, see Additional file 1: Figure S11) that are heterozygous in the proband but homozygous in both IBD segments so that we are confident that each IBD segment will be placed into parental groups correctly with respect to the other IBD segments. Under this constraint, it is straightforward to build subclusters and each will overlap part or all of a chromosome.

Once the subclusters are defined, and all IBD segments within them are separated into two groups, the next step is to align the subclusters’ parental groups and combine them into larger “superclusters”. If the proband shares DNA with the same database individual on two different IBD segments in two different subclusters, we prefer to build a supercluster that puts both of these IBD segments in the same parental group. Our objective is to maximize the number of such instances. In other words, a connection between two subclusters consists of a pair of IBD segments that represent two places where DNA is shared between the proband and the same database individual. A supercluster is a connected component of subclusters. Each subcluster consists of two groups of segments, each representing a different parent of the proband. Our goal is to assign one parent to each of the groups in each subcluster that maximizes the number of subcluster connections between groups assigned to the same parent (see Additional file 1: Figure S1). In practice, to break ties and to weight more informative closer relations higher, each connection is weighted by the amount of DNA shared with the proband.

We choose a simple greedy optimization approach with random restarts to align the parental groups of subclusters which is efficient and works well in practice (See Additional file 1: Figure S1). We begin by grouping subclusters into connected components (so we do not jointly process disjoint sets of subclusters) For each component, we begin by phasing the subclusters randomly (i.e., align *sub*cluster parent *A* randomly with *super*cluster parent *A* or *B*), then greedily swapping the assignments of whichever subcluster increases the objective score the most (i.e., the magnitude of connections between subclusters such that the proband shares an IBD segment in two different subclusters and they are assigned to the same supercluster parent group). We repeat the random-initialization and greedy optimization some number of iterations (1000) and keep the best subcluster phase (i.e., the one with the highest overall objective score).

We are not guaranteed to have enough multi-segment IBD instances to combine all subclusters (some subclusters may have zero connections to others). And, in our experiments after carrying out the procedure above, we remove subclusters from a supercluster unless it has a minimum number of connections to the rest of the supercluster (3). This is done to ensure that a supercluster is a strongly connected component and does not affect the genome-wide phasing result (which must use all subclusters if they are connected or not), but does affect the supercluster phasing and segment labeling accuracy (Additional file 1: Figures S4, S7, and S13, and Table S5). In our experiments, the majority of IBD segments belong to the largest supercluster (96% on average).

Once IBD segments are assigned to superclusters and their parental groups, we phase the proband across the entire genome. We choose to do so at this point because (i) we have now resolved the phase of potentially overlapping subclusters, and (ii) we are interested in examining not just the heterozygous sites in the proband, but also imputing missing data and potentially overriding the original calls if there is enough evidence in overlapping IBD segments to do so. Because the IBD segments are organized into two parental groups, the phased genotype of the proband can be inferred from the homozygous sites in those groups’ overlapping IBD segments. In this step of our approach, we weight segments as we do in step 3 above (see Additional file 1: Figure S11) and consider the weighted sum of IBD segments on either side of the family. If there is enough evidence on either side of the family, IBDphase will override the original calls if necessary to be consistent with overlapping IBD segments. If there is not enough overlapping IBD evidence, IBDphase relies on the call (and the phase, for heterozygous SNPs) made by the pre-phased version of the proband. In our experiments, IBDphase considers 1.0 weighted segments sufficient to override a genotype call, and 0.1 weighted segments sufficient to override an imputed call (i.e., if the call is missing from the proband genotype data and imputed in the pre-phased data).

### Estimating phase confidence

A minority of individuals in our test set are phased inaccurately, with genome-wide phase accuracy as low as 50% (i.e., the alleles inherited from each parent are evenly mixed throughout the genome). These individuals may have accurate local phase, but our goal is to separate the DNA inherited from each parent on a genome-wide scale. The genome-wide phase accuracy depends on the ability of IBDphase to identify the DNA inherited from the same parent in phased data (our subclusters) across different chromosomes and that accuracy is not a function of the number or distribution of IBD segments that applies in all cases.

To identify low-performing cases, we build a random forest regression model that uses the following features.

- *Close family* the number of IBD relationships sharing at least 400 cM that can be identified as being on only one side of the family because they do not share any detected IBD with all of the other individuals that share at least 400 cM of IBD with the proband.
- *IBD statistics* number of database individuals with whom IBD is identified, proportion of those with multiple IBD segments, and proportion of those with exactly one segment.
- *Clustering features* number of IBD segments linked to the largest supercluster, number of database individuals assigned to opposite parents within that supercluster, mean and median ratio between the majority and minority parent side that IBD segments are assigned to.
- *IBD genome coverage* the proportion of the genome that overlaps a minimum number of IBD segments. One feature for each of 1$\times$, 2$\times$, 5$\times$, 10$\times$, and 20$\times$ coverage.

We train the error model on a separate set of 25,641 trios (i.e., these are not the 30,000 individuals used to generate the results above), and use a set of 6,410 trios for validation. We train our model using *scikit-learn* [10] with a parameter grid to optimize the model, which we use to predict the accuracy of the parent assignment to IBD segments in the largest supercluster in the validation set. The relationship between predicted and observed phase accuracy on the test set of 30,000 is shown in Additional file 1: Figure S9.

In practice, we may use the estimated error to make more conservative judgements about our IBD segment clusters. For example, if the predicted error is over 20% (which is the case for about 5% of individuals), we replace the largest supercluster with the largest *sub*cluster, expressing a higher degree of confidence on a smaller portion of the genome, before calculating the accuracy in Additional file 1: Figures S7 and S13.

### Experimental methodology

Our database consists of approximately 12.7 million individuals who have consented to be included in this study. For all our experiments, we limit these data to 416,176 autosomal SNPs, which represents the intersection of SNPs on the autosome that have been reliably called on the various arrays used to genotype these data between 2012 and 2022.

We use the following procedure to carry out all of our experiments. We select 30,000 parent-parent–child trios (90,000 unique individuals) from our database by identifying triples such that the child shares DNA on one haplotype across approximately 100% of the autosome with both parents, but the parents do not share DNA with each other (the parents may share up to approximately 400 cM, which still makes parent-parent–child the only feasible relationship scenario). We select trios uniformally at random from those that can be identified within our database without allowing any test set individual to be included more than once as either child or parent.

We identify IBD shared between members of this test set and the rest of our database. There are several available methods for identifying IBD [11–14] but for the purposes of our experiments we define an IBD segment to be any section of a chromosome of significant length such that two individuals share at least one allele throughout the segment (see Henn et al. [8] for an analysis of the accuracy of IBD defined this way) We consider an IBD segment to be of significant length if (i) it is at least 8 centimorgans, and (ii) it measures least 5 centimorgans when we discard any recombination distance between two adjacent SNPs that exceeds 0.05 centimorgans (the second criterion is designed to ensure that any IBD segment we use is supported by a minimum SNP density). We identify these IBD segments using unphased data, which has the advantage of not relying on any properties of phasing quality at all, though IBDphase does not rely on the particular method for identifying IBD segments. We identify IBD segments in unphased data efficiently by first organizing all genotype data into bitmaps that represent the SNPs where individuals are homozygous for either allele, then comparing the genotypes of pairs of individuals over several SNPs simultaneously using bitwise arithmetic. The basis for our procedure is illustrated in Additional file 1: Figure S12.

Before we run IBDphase, we generate a “pre-phased” version of each trio child in our test set that will be the proband in the experiments. We emphasize that this pre-phasing step does not affect the assignment of IBD segments to either side of the family, and only influences the phased results in positions where IBD segments do not have the homozygous genotype data that determine the phase. However, without such a phased version to default to, each such site would be phased no better than random guessing. For this step, we use Eagle v2.4.1 [6] and provide only the cohort of 30,000 test set trio child individuals.

We provide the pre-phased version of each trio child, the unphased genotype database (including those of the test set), and the IBD segments for each trio child (with those segments shared with parents removed from the list) to IBDphase, which produces the genome-wide phased version of each test set child, as well as the subcluster, supercluster, and parental side labels for each IBD segment. Subclusters consist of IBD segment groups such that each segment in a subcluster overlaps with another segment in the same subcluster by at least 40 sites that are heterozygous in the proband and homozygous in both IBD segments. IBDphase will break up segments if different parts of them are assigned to different parents, but the longest portion of all IBD segments is always retained for the purpose of evaluating their parental side assignment, and all segment portions greater than 5 centimorgans are retained (if multiple 5 cM segment portions are separated by only a single SNP, they will be considered one segment).

To evaluate the results, we introduce the genotypes of the parents in our test set and measure the proportion of heterozygous genotypes in the trio children that are phased in agreement with the genotypes of the parents. Note that the genome-wide phase that results from IBDphase has its alleles divided into two genome-wide haplotypes, but does not associate either specifically with the proband’s mother or father. We consider the genome-wide phase to be accurate if haplotype one agrees with the genotypes of the mother and haplotype two with the father, or if haplotype two agrees with the mother. In this step, we consider all SNPs where the correct phase can be inferred from the parents’ genotypes (i.e., at least one parent is homozygous) and consider the error to be the number of alleles assigned incorrectly allowing for either haplotype-parent assignment, whichever is the better agreement. We also assign a parent side to each IBD segment and compare those to the IBD segments of the parents themselves (See Additional file 1: Figure S12). Unlike global phase accuracy, this measure cannot penalize for parts of the genome that are incorrectly phased but have no IBD segments to inform those sites.

For the imputation and error correction experiments, we perturb the genotypes of the test set trio children before the IBD detection step by replacing 1% of the calls (selected uniformally at random) with missing data and changing the genotypes for 0.2% of the calls either from heterozygous to homozygous or from homozygous to heterozygous. In our experiments, IBDphase infers the genotype of the proband from those of overlapping IBD segments as long as there is at least one overlapping segment (after downweighting the SNPs near the segment’s endpoints by the sigmoid-shaped weighting function described above and shown in Additional file 1: Figure S11) and overrides the imputed call or phase decision given by the pre-phased data if the count of weighted IBD segments exceeds 0.1. Note that if we perturb the data such that we change a heterozygous SNP in the proband to homozygous opposite the homozygous genotype of another database individual with which the proband shares an IBD segment, our IBD detection procedure will not include the segment. Therefore, to evaluate IBDphase for identifiying and correcting genotype errors, we detect IBD using a subset of SNPs (about 63% of the original set, selected uniformally across the genome), and observe whether IBDphase imputed or replaced calls correctly on the SNPs that were not used in IBD detection (although the results are almost identical when we evaluate using all SNPs; see Additional file 2).

## Supplementary Information

**Additional file 1.** Supplementary Figures, Tables, and Appendecies.

**Additional file 2.** Supplementary Data Tables.

## Data Availability

All data generated for this study are available in Additional file 2. Individual genotype data for human subjects participating in Ancestry DNA’s Human Diversity Project are not available, to protect their privacy and anonymity.

## Abbreviations

cM: Centimorgan
IBD: Identity by descent
SNP: Single nucleotide polymorphism
