# Supplementary material for: Accurate genome-wide phasing from IBD data
Source: BMC Bioinformatics. 2022 Nov 23;23:502. doi: 10.1186/s12859-022-05066-2 (PMC9686111; doi:10.1186/s12859-022-05066-2)
Supplement: Supplementary file 1 — Additional file 1. Supplementary Figures, Tables, and Appendecies. [file 12859_2022_5066_MOESM1_ESM.pdf]

Additional File 1

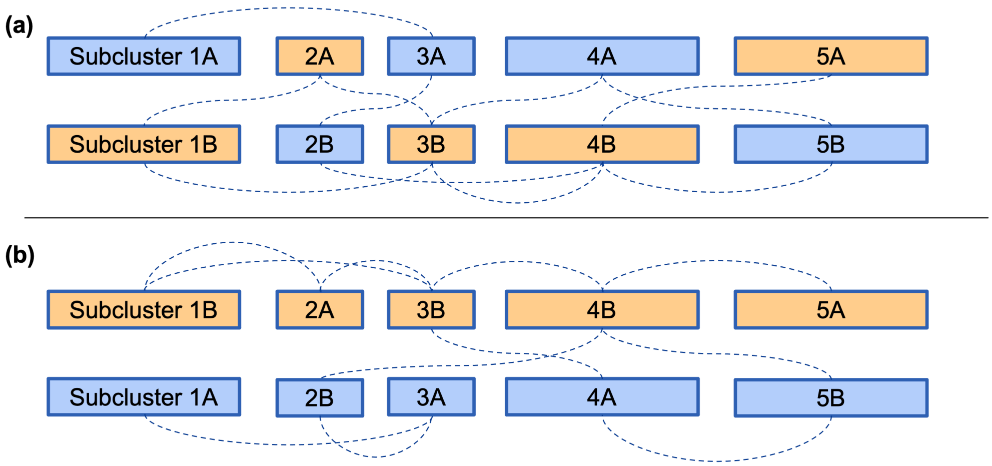

S1 Fig. An illustration of the subcluster phasing task. **(a)** shows five subclusters numbered 1-5 across four chromosomes. The color indicates the true parent from which IBD segments are inherited. The letter *A* or *B* indicates which side of the family each subcluster was originally assigned. Dotted lines indicate a connection between groups where the proband shares DNA with the same person in both groups. **(b)** shows the result of phasing these subclusters into one supercluster such that the number of connections between groups on the same side of the family is maximized.

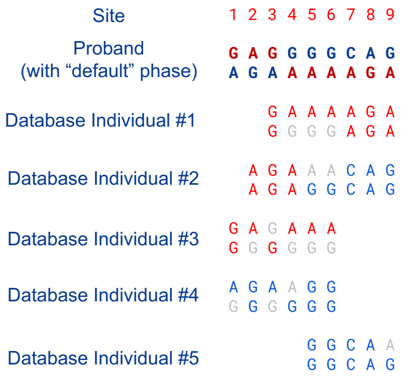

	Site #1	Site #2	Site #3	Site #4	Site #5	Site #6	Site #7	Site #8	Site #9
Parent group A	#3	#2, #3	#1, #2, #3	#1, #2, #3	#1, #2, #3	#1, #2, #3	#1, #3	#1, #3	#1, #3
Parent Group B		#4	#4	#4	#4, #5	#4, #5	#2, #4, #5	#2, #4, #5	#2, #4, #5
Parent Group Not Assigned	#1, #2, #4, #5	#1, #5	#5	#5					

At site 1: only individual #3 is homozygous. Keeping with “default” phase, add to parental group A.
At site 2: #2 and #4 are homozygous for opposite alleles. Keeping with “default” phase, add #2 to A, #4 to B.
(since allele “A” follows “G” as the first two alleles in the same haplotype of the pre-phased “default” phase, and allele “G” was added to group A at site 1, add database individual #2 with an “A” at site 2 also to group A.)
At site 3: #1, #2, #3 are homozygous. Rather than moving #2 and #3 to group B, we add #1 to A.
At site 4: only #2 is homozygous. No change.
At site 5: #4 and #5 are homozygous for the same allele. Add unassigned #5 to the same group as #4, group B.
At site 6: #4 and #5 are homozygous for the same allele. No change.
At site 7: #1 is homozygous A, #2 and #5 are homozygous C.
Move one segment (#2 to group B) rather than moving two segments (#1 to B and #5 to A).
At site 8: #1 and #2 are homozygous for opposite alleles. No change.

S2 Fig. An illustration of the IBD segment assignment task. This toy example shows nine sites where the proband is heterozygous, the “default” phase (*e.g.*, provided by a phasing model), and five IBD segments. Moving from left to right, we can group the IBD segments into two parental groups based on their homozygous sites: segments that are homozygous for the same allele belong to the same parental group. In this example, there is no way to consistently assign all segments to one group or the other (at site 7, individual #2 who was previously grouped with individual #1 is transferred from parental group A to B).

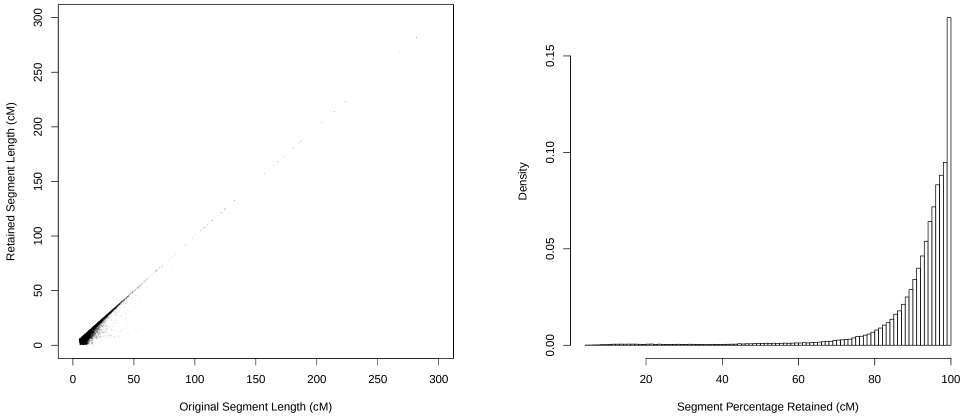

S3 Fig. Proportion of segment length retained after assigning parental groups. Both plots show the same data indicating how much of an original IBD segment's length is retained after the process of assigning each to a parental group (the sample mean is 92% and the median is 96%.) To resolve Mendelian confilicts, some segments must be broken.

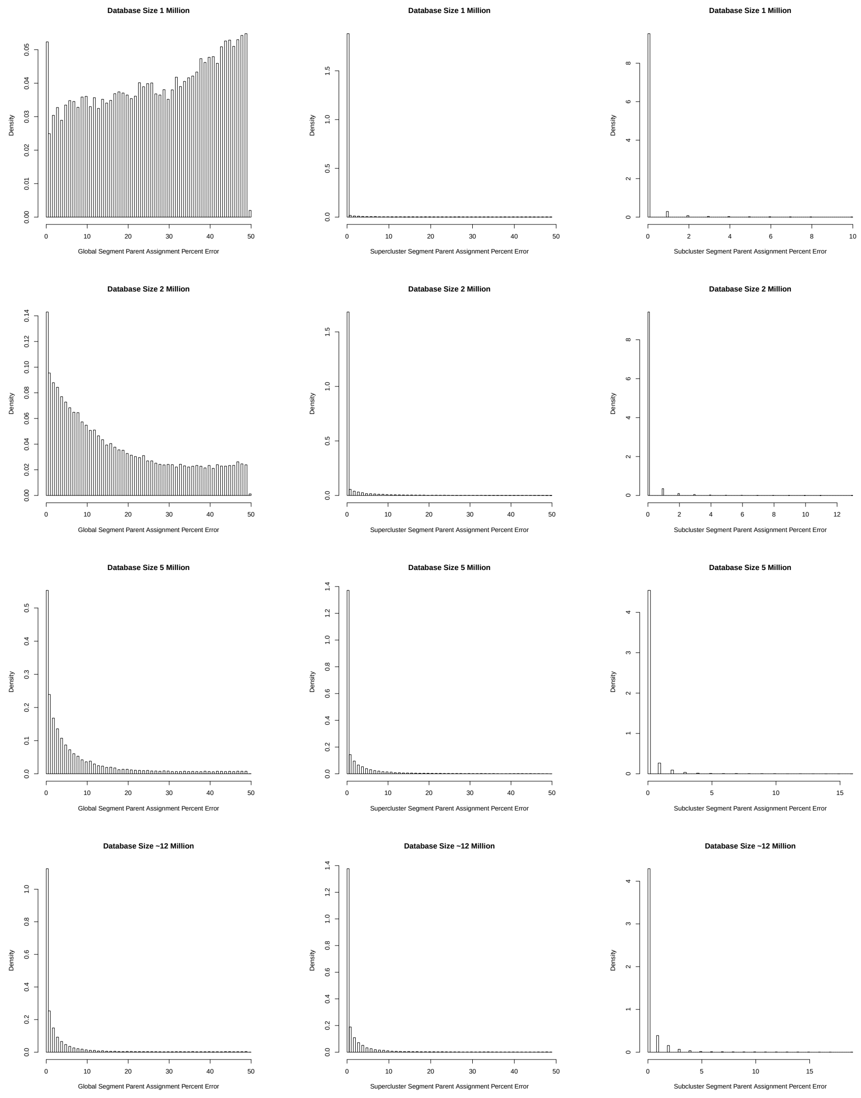

S4 Fig. Distribution of phase error based on the size of the database against which we search for IBD. Column 1 shows the global phase error. Column 2 shows the aggregate phase error when each supercluster is evaluated independently. *I.e.,* if two superclusters both separate maternal and paternal alleles well, they are both evaluated with low error, even if they disagree on which parent is which. Column 3 shows the aggregate phase error when each subcluster is evaluated independently. Only SNPs that belong to IBD segments are used in the evaluation in columns 2 and 3 which include 90.0% of SNPs when the database size is 1 million, 95.1% at 2 million, 98.9% at 5 million, and 99.6% of SNPs in the full database of ∼12 millon. (Note that the horizontal range of column 3 differs from the others.)

**a.** Global phase error rate	Mean	Median
Pre-Phased	0.4810	0.4842
IBDPhase	0.0493	0.0209
IBDPhase (largest supercluster)	0.0493	0.0209
IBDphase (SNPs covered by IBD segments)	0.0472	0.0195
IBDphase (largest supercluster, covered by IBD segments)	0.0302	0.0118

**b.** Switch error rate	Mean	Median
Pre-Phased	0.0124	0.0108
IBDPhase	0.0033	0.0026
IBDPhase (largest supercluster)	0.0033	0.0026
IBDphase (SNPs covered by IBD segments)	0.0032	0.0026
IBDphase (largest supercluster, covered by IBD segments)	0.0027	0.0023

**c.** Length (cM) of runs of correct phase	Mean	Median
Pre-Phased	2.8057	2.8743
IBDPhase	13.4194	11.2563
IBDPhase (largest supercluster)	13.4194	11.2563
IBDphase (SNPs covered by IBD segments)	13.6185	11.4408
IBDphase (largest supercluster, covered by IBD segments)	17.0442	14.0138

**d.** Proportion of SNPs in 1 cM+ runs of correct phase	Mean	Median
Pre-Phased	0.9368	0.9505
IBDPhase	0.9864	0.9901
IBDPhase (largest supercluster)	0.9864	0.9901
IBDphase (SNPs covered by IBD segments)	0.9877	0.9904
IBDphase (largest supercluster, covered by IBD segments)	0.9921	0.9926

S5 Table Phase accuracy statistics. Accuracy of phase when (i) Pre-phased (*i.e.,* using Eagle alone on the 30,000-member test set), (ii) using IBDphase, (iii) using IBDphase, but limited to SNPs in the largest supercluster, (iv) using IBDphase, but limited to SNPs that are covered by at least one IBD segment, (v) using IBDphase, but limited to SNPs that are both in the largest supercluster and covered by at least one IBD segment. The accuracy is showed as measure by (**a.**) Global phase error rate: what proportion of heterozygous SNPs are phased in a way that agrees with trio phase (only counting SNPs that are unambiguously phased by the parents–*i.e.,* when at least one parent is homozygous), (**b.**) Switch error rate: what proportion of heterozygous SNPs are phased opposite the previous heterozygous SNP, relative to the trio phase? (**c.**) Average length (cM) of runs of correct phase: what is the average number of centimorgans before a switch error is encountered? (**d.**) Proportion of SNPs in 1 cM+ runs of correct phase: how many SNPs are in runs of correct phase that are at least 1 centimorgan?

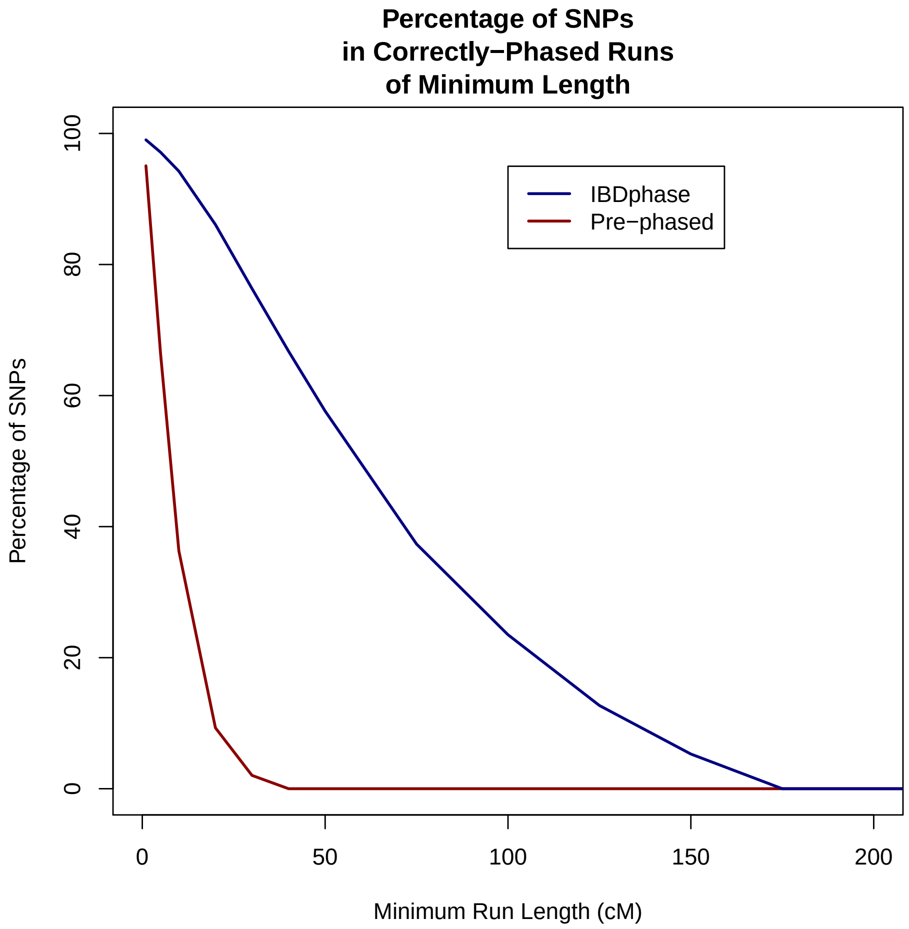

S6 Fig. Length of Runs of Verified-Correct Phase. Distribution of the median proportion of SNPs that are part of runs of various minimum length that are in 100% agreement with the trio-phased standard. For example, a majority of SNPs phased with IBDphase are part of runs of at least 50 centimorgans that are identical to trio phase.

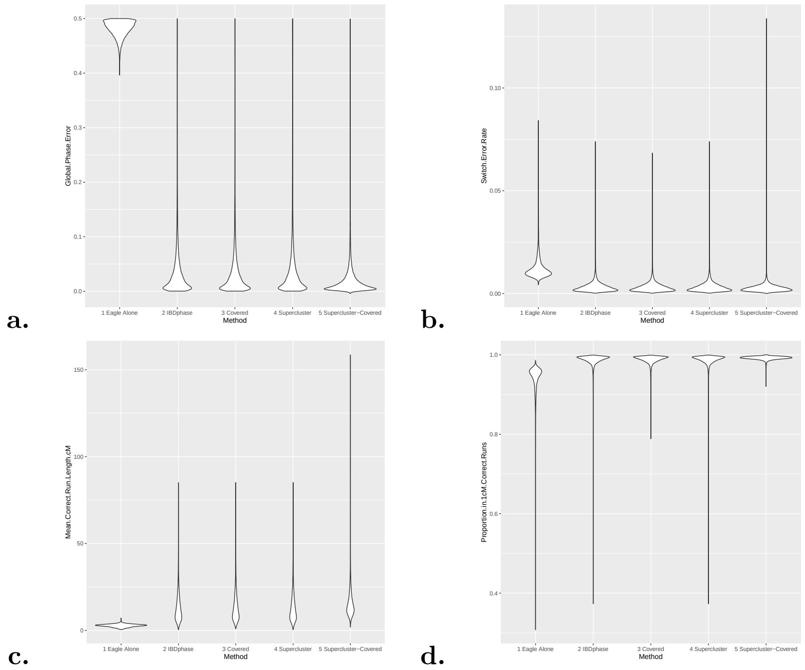

S7 Fig. Distribution of phase error. Violin plots showing the distribution of phase error rates measured in various ways on (1) data phased with Eagle alone, (2) data phased with Eagle, then IBDPhase, (3) IBDPhase restricted to SNPs that are covered by at least one IBD segment, (4) IBDPhase restricted to sections of the genome that overlap the largest supercluster, (5) IBDPhase restricted to the largest supercluster and are covered by an IBD segment. **a.** Distribution of global (genome-wide) phase error: assuming we pair each haplotype with whichever parent data is the most favorable, how many alleles in heterozygous sites are assigned to the wrong parent? We calculate this by comparing the phase with the parents' genotypes at sites where the correct phase is unambiguous (*i.e.,* at least one parent is homozygous). **b.** Distribution of switch error rate: what proportion of (unambiguously phased) heterozygous sites are out-of-phase with the previous heterozygous site? **c.** Distribution of the average length of runs of correct phase: what is the mean length (in centimorgans) of a section of the genome that is uninterrupted by a phase switch error? **d.** Distribution of the proportion of the genome that is in a run of correct phase that is at least 1 cM long: what proportion of (unambiguously phased) SNPs are in runs of at least 1 cM that are uninterrupted by a phase switch error?

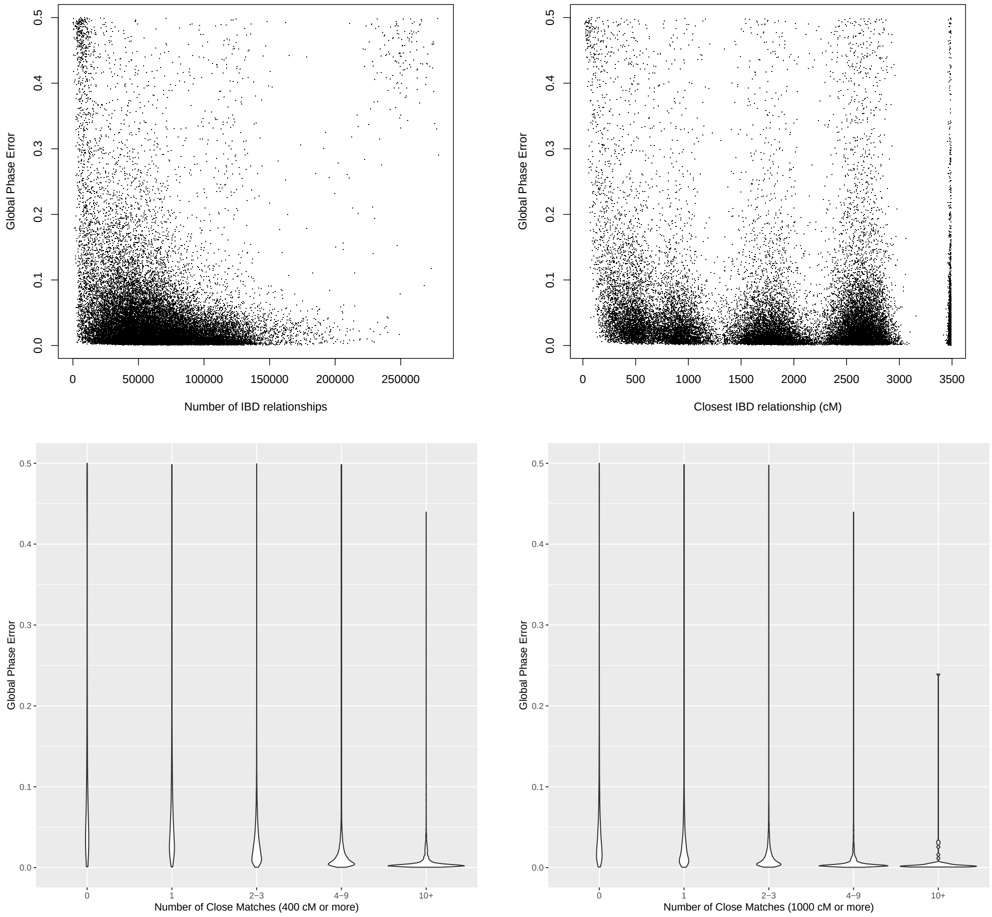

S8 Fig. Plots comparing IBD statistics and global phase accuracy. The top two scatterplots show that there is no strong relationship between the number of IBD segments nor the closest genetic relationship, and the accuracy of phase. However, the number of close relationships clearly have a positive correlation with accuracy, as shown by the bottom two violin plots.

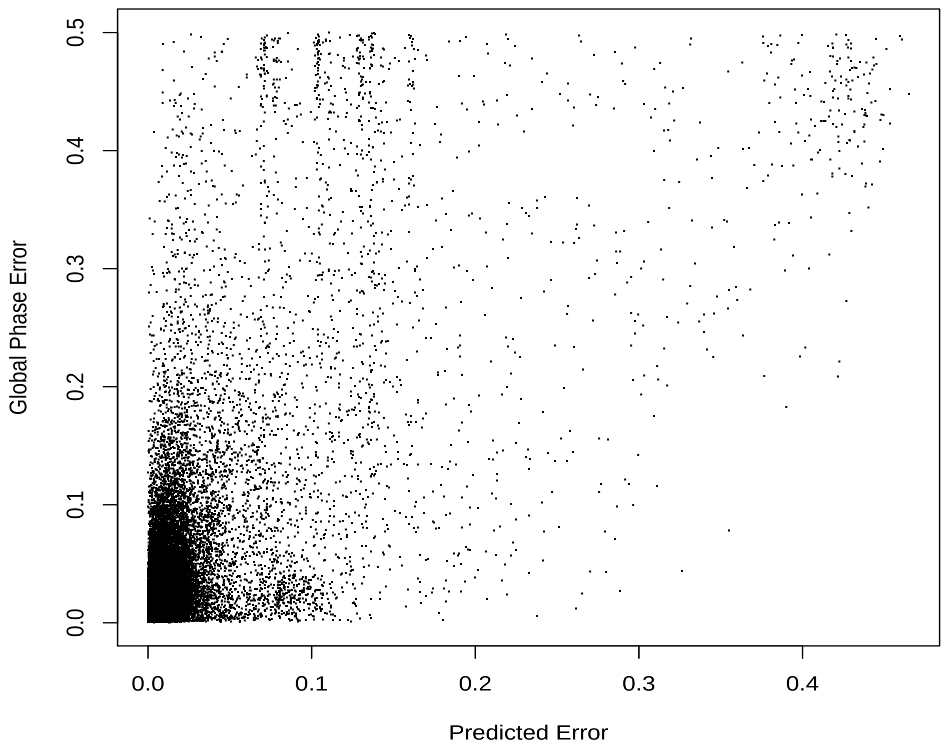

S9 Fig. Scatterplot comparing predicted IBD clustering error and global phase accuracy.

Region	Median Global Phase Error	Median Proportion at 5x Coverage	Sample Size	Median Global Phase Error Among Those with 90% 5x Coverage	Sample Size
Western Europe	0.019	0.988	25980	0.018	24505
Middle East	0.020	0.947	40	0.013	31
Northern Europe	0.020	0.980	1328	0.019	1250
West Africa	0.022	0.962	596	0.019	528
European Jewish	0.034	0.995	522	0.034	518
Southeast Asia	0.040	0.904	59	0.023	31
Central America	0.050	0.984	346	0.039	305
Eastern Europe	0.066	0.916	949	0.036	536
East Asia	0.070	0.904	47	0.052	24
Polynesia	0.093	0.985	31	0.057	29
South Asia	0.266	0.704	20	0.077	5
South America	0.349	0.640	37	0.029	3

S10 Table Performance Across Various Database Demographics. Each individual is classified as whichever group AncestryDNA estimates explains the origin of more DNA than any other. Global phase error is shown with all such individuals, and after limiting to those with 90% of the genome overlapped by 5 or more IBD segments.

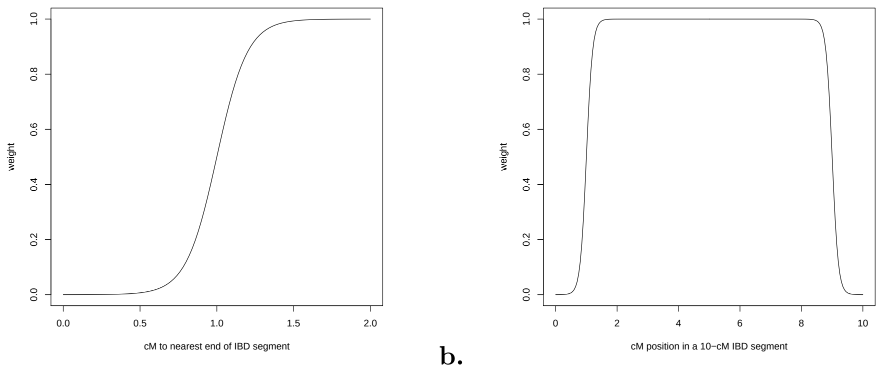

a. **b.**

S11 Fig. The weight function that we overlay on IBD segments when making phasing decisions based on the genotypes of multiple IBD segments. The function is $weight = (1 + e^{-10\times(cM-1)})^{-1}$ where cM represents the distance in centimorgans to the nearest end of the IBD segment. The motivation for doing this is that it is difficult to pinpoint where DNA sharing because of IBD begins and ends, and therefore the information nearest the ends is less reliable.

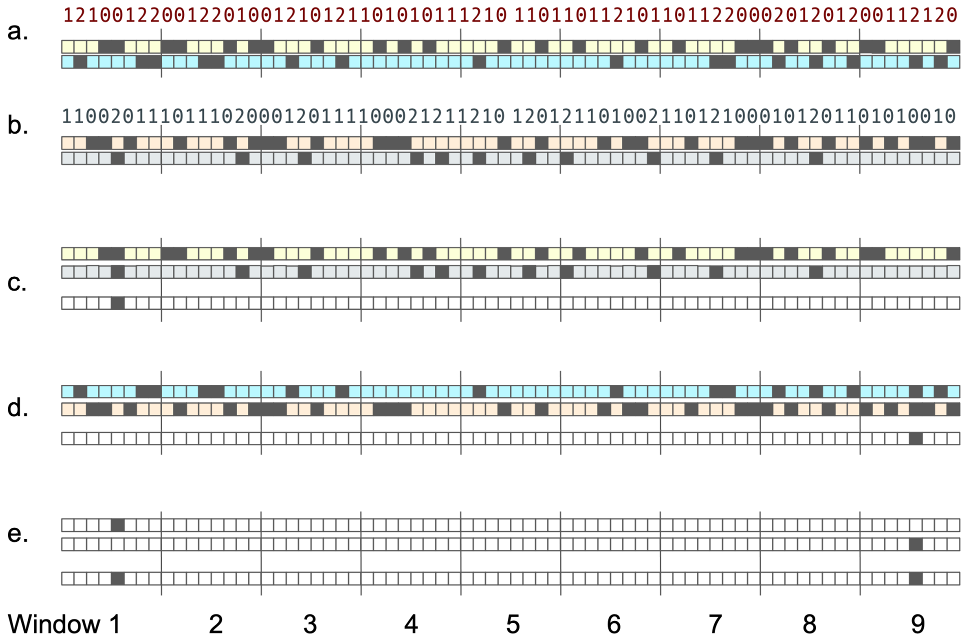

S12 Fig. Illustration of the bitwise arithmetic used to discover IBD segments. Part **a.** shows the genotype of one individual, A, expressed as the number of copies of an alternate allele, 0, 1, or 2, and two corresponding bitmaps: the positions where the individual is homozygous for the reference allele, and the positions where the individual is homozygous for the alternate allele. **b.** shows the same for another individual, B. **c.** shows the result of applying bitwise *and* to individual A's homozygous-reference bitmap and B's homozygous-alternate bitmap. **d.** shows the result of applying bitwise *and* to individual A's homozygous-alternate bitmap and B's homozygous-reference bitmap. **e.** shows the result of applying bitwise *or* to the results from **c.** and **d.** The result in **e.** tells us that individuals A and B do not share an allele for one of the SNPs in window 1 because that window evaluates to nonzero, but there are no such homozygous opposites in windows 2-8. If that segment is long enough, we can compute the exact coordinates of the IBD segment. (The illustration uses 8-bit/8-SNP windows, but in practice we use 64-bit arithmetic.)

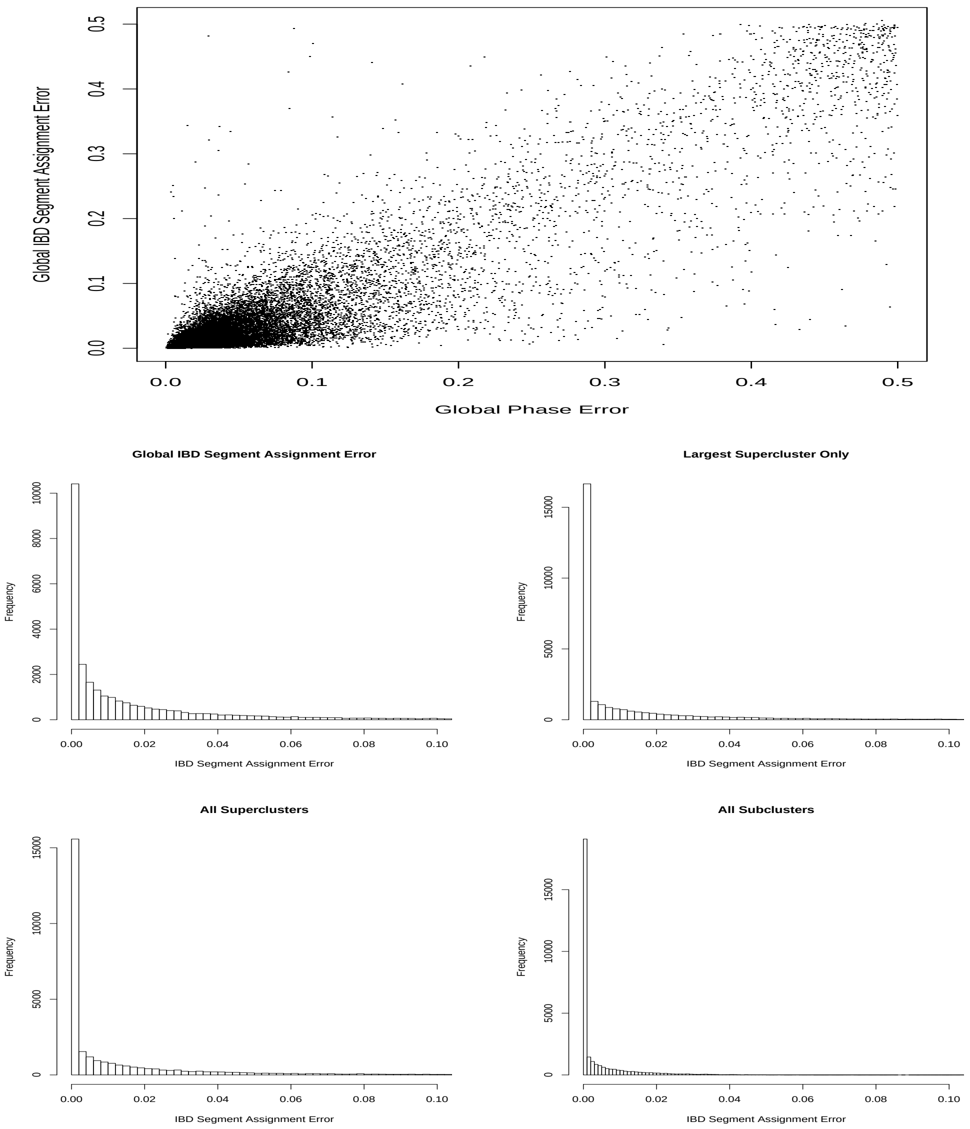

S13 Fig. **IBD Segment Assignment Error.** These plots show the accuracy of assigning the IBD segments of a test set trio child to two parents, where accuracy is determined by whether the same IBD segments are also found in the IBD data of the parents. The first plot compares this measure of accuracy to global phase accuracy. The others show histograms of error distribution if we include all IBD segments, just the ones in the largest supercluster of each test set example, if we evaluate each supercluster independently of the others, and if we evaluate each subcluster independently of the others.

Cohort Size	Phasing Software	Switch Error Rate	Runtime (Avg. per Test Set Indiv.)
test set only (cohort of 30K)	BEAGLE (v. 5.4)<br>Eagle (v. 2.4.1)<br>SHAPEIT4	0.013<br>0.020<br>0.016	0.027 seconds<br>0.14<br>0.074
test set + 70K (cohort of 100K)	BEAGLE (v. 5.4)<br>Eagle (v. 2.4.1)<br>SHAPEIT4	0.0065<br>0.011<br>0.0078	0.086<br>0.45<br>0.18
test set + 470K (cohort of 500K)	BEAGLE (v. 5.4)<br>Eagle (v. 2.4.1)<br>SHAPEIT4	0.0024<br>0.0049<br>0.0031	0.46<br>3.5<br>0.74
test set + 970K (cohort of 1M)	BEAGLE (v. 5.4)<br>SHAPEIT4	0.0016<br>0.0022	0.86<br>1.4
12.7M (IBD)	IBDphase	0.0067	29

S13 Table Local phase accuracy statistics on chromosome 22. Measurements of the local phase accuracy, measured as the proportion of consecutive heterozygous SNPs that disagree with trio phase, considering only SNPs where the trio phase is unambiguous (at least one parent is homozygous). SHAPEIT4, BEAGLE, and Eagle were run on chromosome 22 data only (6,243 SNPs). The table also shows average runtime per *test set* individual (if the cohort is larger, the runtime is still measured as total-process-runtime divided by 30,000). IBDphase was run on the entire genome (by design), but the local phase results shown are based on the same 6,243 chromosome 22 SNPs as the other approaches. (Note that our run of Eagle with a cohort of 1M timed out after 72 hours and results are not shown.)

Minimum IBD Considered	Median Genome-Wide Phase Error	Mean Phase Error	Runtime (Estimated Avg. Per Test Indiv.)
5cM	0.037	0.067	89 seconds
6cM	0.036	0.065	57
7cM	0.026	0.055	35
8cM	0.021	0.049	23
10cM	0.018	0.048	14
12cM	0.019	0.049	9.2
15cM	0.021	0.052	5.7
20cM	0.027	0.063	3.2

S14 Table IBDphase Genome-wide phase accuracy statistics varying the minimum IBD used. These data were generated from a subset of **3,000** (selected uniformily at random) of the test set of 30,000.

A Pseudocode

The following pseudocode correpsonds to the implementation of IBDphase as described in the Methods Section.

Algorithm 1 Perform IBDphase on one proband. P is proband information, M is IBD data.

```
procedure IBDphase(P,M)

    // Step 1: Load genotype data from disk,
    // store along with the IBD segment data in M
    load_genotype_data(P,M)

    // Step 2: Compare close family to see if they are related,
    // add relationship status to the close family individuals' IBD data in M.
    analyze_close_family(M)

    // Step 3: Separate overlapping IBD into two parental groups,
    // assigning segments to either group 1 or group 2 at a local level
    separate_overlapping_IBD(P,M)

    // Step 4: Define groups of overlapping IBD. Group overlapping segments
    // (there will be one group per chromosome, or a few if the whole chromosome
    // doesn't have continuously overlapping IBD throughout)
    create_subclusters(P,M)

    // Step 5: Combine subclusters into superclusters, connected by
    // instances of IBD shared with the same person in different
    // subclusters. Phase each of these connected components (superclusters)
    // so that parental groups 1 and 2 are the same parents in all subclusters
    create_superclusters(M)

    // Step 6: Use the segments' supercluster parental group assignment
    // and genotype data to assign phase to the proband
    phase_proband(P,M)
```

Algorithm 2 Load genotype data from disk and associate with each segment of IBD in the input IBD data, M. To be efficient, it is essential that genotype data are stored in binary format and loaded directly from a file position based on the genomic coordinates of each IBD segment. In our experiments, we used 400cM as the close relation threshold.

```
procedure LOAD_GENOTYPE_DATA(P,M)
    load pre-phased whole-genome genotype data for proband, add to P
    load unphased whole-genome genotype data for proband, add to P
    for each m ∈ M do // for each database individual that shares IBD with proband
        if m.cM ≥CLOSE_RELATION_THRESHOLD then // we used 400 cM as the threshold
            load genotype data g of whole genome, add to m
        else
            for each s ∈ m.segments do // for each IBD segment
                load genotype data g within coordinates of s, store data along with s
```

Algorithm 3 Checks to see if pairs of the proband's relatively close relationships are related to each other, and use that information to mark those individuals as either (i) on one side of the family or the other, or (ii) potentially a descendent of the proband's parents, who would share IBD on both sides of the family. The function ESTIMATE_IBD is not defined in pseudocode, but it measures runs of IBD along the genome. In our experiments, we used 400cM as the close relation threshold, and 20cM as the minimum IBD sharing threshold (if two database individuals that share IBD with the proband share 20cM with each other, they are not assumed to be on different sides of the proband's family).

```
procedure ANALYZE_CLOSE_FAMILY(P,M)

    // gather individuals' data in M that are relatively close family
    // (in practice, this is created in LOAD_GENOTYPE_DATA)
    F ← {m ∈ M|m.cM ≥CLOSE_RELATION_THRESHOLD} // we use 400cM

    N ← {} // close family network to populate
    for each pair (a, b) ∈ F do // a,b are both close family of proband
        cM ←ESTIMATE_IBD(a.g, b.g)// estimate IBD between a and b
        if cM ≥ MIN_IBD_THRESHOLD then // we use 20cM, are a and b related?
            add edge (a, b) to N
    for a ∈ F do
        if a.relation is SIBLING or TWIN then // individual a's relation to proband
            mark a as such
        else if ∀b ∈ F, (a, b) ∈ N then // if a shares DNA with all proband's close family
            mark a as a possible descendent of proband's parents
        else// ∃b ∈ F, (a, b) ∉ N
            mark a as being on one side of the family only
```

Algorithm 4 Assign a weight to an IBD segment s relative to a genomic position m. This weight will be the "cost" of breaking the segment when assigning multiple segments to parental groups. Assign low weights to segments where m is near the beginning or end of the segment, where there is less certainty of a shared haplotype.

```
procedure WEIGHT(s,d)
    // Compute weight of segment s relative to position (SNP) d
    // (See Figure S11)
    if s.status ∈ {TWIN,SIBLING,POSSIBLE_DESCENDENT} then
        return 0 // don't worry about breaking up segments we suspect to be on both sides
    else
        x ← min(s.end − d, d − s.begin) // distance to nearest segment endpoint
        return 1 + 1/e^(−10×(x−1))
```

Algorithm 5 Compare the genotypes of overlapping IBD segments and separate them into two parental groups. If two IBD segments are homozygous opposites at the same site, they belong in different groups. Resolve conflicts by breaking up IBD segments but break as few as possible with a preference for breaking them near the original segment enpoints, preserving as much of the segment as possible. If we find that we must break many segments at a site, yet ignoring the site doesn't require breaking many segments at nearby sites, we will ignore the site (*i.e.*, chalk it up to a genotype error)

```
procedure SEPARATE_OVERLAPPING_IBD(P,M)
    for each chromosome c do
        G_1 ← {}, G_2 ← {} // two parental groups, initially empty
        for each SNP d on chromosome c that is heterozygous in the proband do
            A_0 ← all IBD segments in M that contain SNP d and are homozygous for
                    allele 0 at SNP d
            A_1 ← all segments homozygous for allele 1 at d
            C_{A0G1} ← Σ_{s∈(A_0∩G_2)∪(A_1∩G_1)} WEIGHT(s,d) // cost of assigning A_0 to G_1
            C_{A0G2} ← Σ_{s∈(A_0∩G_1)∪(A_1∩G_2)} WEIGHT(s,d) // cost of assigning A_0 to G_2

            if min(C_{A0G1}, C_{A0G2}) ≥ HIGH_COST_THRESHOLD then // we use 1.0
                // High cost. Could be a genotype error in the proband.
                // Consider ignoring this site and maintaining groups as they are

                G'_1, G'_2 ← G_1, G_2 // Groups assuming we use the SNP d
                // break up the segments we must based on the genotypes at d
                if C_{A0G1} ≤ C_{A0G2} then
                    ASSIGN_PARENTAL_GROUPS(G'_1, A_0, G'_2, A_1, d)
                else
                    ASSIGN_PARENTAL_GROUPS(G'_1, A_1, G'_2, A_0, d)
                cost_keep ← LOOKAHEAD_COST(G'_1, G'_2, d, L, P, M, UPSTREAM)
                          + LOOKAHEAD_COST(G'_1, G'_2, d, L, P, M, DOWNSTREAM)

                G''_1, G''_2 ← G_1, G_2 // Groups assuming we ignore the SNP d
                // (don't break up these segments based on SNP d)
                cost_ignore ← IGNORE_SNP_BASE_COST // we use cost=1
                            + LOOKAHEAD_COST(G''_1, G''_2, d, L, P, M, UPSTREAM)
                            + LOOKAHEAD_COST(G''_1, G''_2, d, L, P, M, DOWNSTREAM)

                // ignore the SNP d iff the cost to keep it is higher, we use 1.0
                ignore ← (cost_ignore < cost_keep and cost_keep / cost_ignore > COST_RATIO_THESHOLD)
            else
                ignore ← FALSE // low cost, don't consider ignoring it


            if ¬ignore then
                // Assign the segments in A_0 and A_1 to groups whichever way has the
                // lowest cost. If the costs are equal, assign the allele to group A_0 that is
                // on the same haplotype in the pre-phased data P as the allele that was
                // assigned to A_0 at the previous heterozygous site
                // (if there is no such site, assign arbitrarily).
                P_{A0G1} ← TRUE if and only if allele 0 at site d is on the same
                        haplotype in P that was previously assigned to group G_1
                if C_{A0G1} ≤ C_{A0G2} (or C_{A0G1} = C_{A0G2} and P_{A0G1}) then
                    // Add segments A_0 to group 1, A_1 to group 2
                    ASSIGN_PARENTAL_GROUPS(G_1, A_0, G_2, A_1, d)
                else
                    // Add segments A_1 to group 1, A_0 to group 2
                    ASSIGN_PARENTAL_GROUPS(G_1, A_1, G_2, A_0, d)

    // now every segment in M has a local-level parent assignment, 1 or 2
```

Algorithm 6 Assign N_1 to parental group G_1 and N_2 to G_2, breaking segments that change groups at SNP d

procedure ASSIGN_PARENTAL_GROUPS(G_1,N_1,G_2,N_2,d)
  for each $s \in (N_1 \cap G_2)$ **do**
    // for each segment s that has to change from G_2 to G_1
    remove s from N_1 and G_2
    break s into two segments, s_u and s_d, splitting at SNP d
    assign s_u to G_2; assign s_d to G_1
  for each $s \in (N_2 \cap G_1)$ **do**
    // for each segment s that has to change from G_1 to G_2
    remove s from N_2 and G_1
    break s into two segments, s_u and s_d, splitting at SNP d
    assign s_u to G_1; assign s_d to G_2
  $G_1 \leftarrow G_1 \cup N_1$ *// add the rest of N_1 to parental group*
  $G_2 \leftarrow G_2 \cup N_2$ *// add the rest of N_2 to parental group*

Algorithm 7 Scan the genome within L sites that are heterozygous in the proband of a SNP d to see how many segments we must break to account for conflicts, and return a measure of the cost that must be incurred.

procedure LOOKAHEAD_COST(G_1,G_2,d,L,P,M,dir)
  $result \leftarrow 0$ *// init. return value, running sum*
  Let H be at most L sites closest to d that are
      (i) heterozygous in the proband, and
      (ii) either all upstream or downstream of d, depending on argument dir,
  for each site $d' \in H$ **do**
    $A_0 \leftarrow$ all IBD segments in M that contain SNP d' and are homozygous for
        allele 0 at SNP d'
    $A_1 \leftarrow$ all segments homozygous for allele 1 at d'
    $C_{A0G1} \leftarrow \sum_{s \in (A_0 \cap G_2) \cup (A_1 \cap G_1)}$ WEIGHT(s, d') *// cost of assigning A_0 to G_1*
    $C_{A0G2} \leftarrow \sum_{s \in (A_0 \cap G_1) \cup (A_1 \cap G_2)}$ WEIGHT(s, d') *// cost of assigning A_0 to G_2*
    // Like SEPARATE_OVERLAPPING_IBD, break segments as necessary
    $P_{A0G1} \leftarrow$ (assigning A_0 to G_1 agrees with P)
    if $C_{A0G1} \leq C_{A0G2}$ (or $C_{A0G1} = C_{A0G2}$ and P_{A0G1}) **then**
      ASSIGN_PARENTAL_GROUPS(G_1,A_0, G_2,A_1, d')
      $result \leftarrow result + C_{A0G1}$
    else
      ASSIGN_PARENTAL_GROUPS(G_1,A_1, G_2,A_0, d')
      $result \leftarrow result + C_{A0G2}$
  return $result$

Algorithm 8 Group segments in M into groups that overlap each other

```
procedure CREATE_SUBCLUSTERS(P,M)
    Let sn ← 0// subcluster serial number, used below

    for each chromosome, c do
        for each segment s in M on chromosome c do
            // construct a set of “informative” sites in this segment, call it I
            s.I ← the set of SNPs in s that are het in P and homozygous in s

        // start with singleton sets, each set is one segment
        Let B ← ({s} for each segment s in M on chromosome c)
        repeat
            for each pair of groups B_i,B_j in B do
                for each pair of segments s_i,s_j, one from each of B_i,B_j do
                    // how many informative SNPs do s_i, s_j have in common?
                    w ← ∑_{d∈s_i.I∩s_j.I} min(WEIGHT(s_i,d),WEIGHT(s_j,d))
                    if w ≥SUBCLUSTER_INFO_THRESHOLD then // we use 10
                        // at least one pair of segments overlap by a convincing enough
                        // number of informative SNPs to combine the groups
                        combine B_i,B_j together, keep in B
        until no groups in B were combined

        // we need only store a unique subcluster number assigned to each segment
        for each group B_i ∈ B do
            Let sn ← sn + 1// unique serial number for subcluster
            for each segment s in B_i do
                assign s.subcluster ← sn

```

Algorithm 9 Given a number of "subclusters", each consisting of a set of segments that are labeled parental side 1 or 2, group subclusters into connected components and phase the subclusters in each component (*i.e.,* choose which parental sides in each subcluster all correspond to the same parent). The objective is to maximize the connections between subcluster parental groups that are placed on the same parental side in the phased result. (See Fig. S1.)

```
procedure CREATE_SUPERCLUSTERS(P,M)

    // 'connections' stores the magnitude of connections between any
    // pair of subclusters a and b, if a's parent 1 and
    // b's parent 1 represent the same parent or different parents
    connections ← SUBCLUSTER_CONNECTIONS(M) // pseudocode below

    // which of those sets of subclusters constitute connected components?
    CC ← CONNECTED_COMPONENTS(connections) // pseudocode below

    for each component B ∈ CC do // B is a set of subclusters
        bestscore ← 0
        for random restart trial ∈ {1, 2, ...,RESTARTS} do // 1,000 times
            assign a binary/Boolean flag B_i.swap randomly to each subcluster B_i in B
            // B_i.swap =TRUE will mean to reverse the parent 1 and 2 labels in b
            // to align with the other subclusters in B

            // compute objective score score
            // also compute delta_i, which will be the positive or negative
            // change (net gain) to the score if we change B_i.swap
            score ← 0 // running sum
            for i ∈ 1, 2, ..., N do // for each subcluster B_i ∈ B
                delta_i ← 0 // initialize delta_i to 0 for each B_i in B
            for i ∈ 1, 2, ..., N do // for each subcluster B_i ∈ B
                for j ∈ i + 1, i + 2, ..., N do // for each other subcluster B_j ∈ B
                    score ← score + connections(B_i, B_j, B_i.swap = B_j.swap)
                    // delta represents both the gains to connections
                    // if we change a B.swap and the losses from connections
                    // we already have with the current settings to all B.swap
                    delta_i ← delta_i + connections(B_i, B_j, B_i.swap ≠ B_j.swap)
                                     − connections(B_i, B_j, B_i.swap = B_j.swap)
                    delta_j ← delta_j + connections(B_i, B_j, B_i.swap ≠ B_j.swap)
                                     − connections(B_i, B_j, B_i.swap = B_j.swap)

            repeat
                i ← argmax_i delta_i // swap B_i maximizes the objective score
                if delta_i > 0 then // increase in objective score
                    // accept the change to subcluster B_i.swap
                    for j ∈ 1, 2, 3, ..., i − 1, i + 1, ..., N do // for all subclusters j except B_i
                        // update delta_j now that B_i.swap will be different
                        // (delta_j already contains the difference (gains and losses)
                        // in objective score we get by changing B_j.swap relative to
                        // B_i.swap. Now that B_i.swap will change, we add or subtract them
                        // twice so that losses become gains and gains become losses.)
                        delta_j ← delta_j +2 × connections(B_i, B_j, B_i.swap = B_j.swap)
                                         −2 × connections(B_i, B_j, B_i.swap ≠ B_j.swap)
                    B_i.swap ← ¬B_i.swap
                    score ← score + delta_i
                    delta_i ← −delta_i // gain would be a loss if we switched B_i.swap again
                else
                    break // exit loop
            until score does not increase

            if score > bestscore then
                bestscore ← score
                bestalignment ← (B_i.swap for B_i ∈ R)

        // now we have our best phasing
        for each B_i, swap_i ∈ B, bestalignment do // "zip" parallel structures
            if swap_i then B_i.parent ← SWAP(B_i.parent) // swap parent number, 1 or 2
```

Algorithm 10 Define connections between subclusters of a set B of N subclusters, each consisting of a set of IBD segments. Each subcluster has two parental groups of segments (call them 1 and 2) and a connection links a parental group in one subcluster to a parental group in another. The result is *connections* where *connections*$(13, 16,$TRUE$)$ represents the connections between subclusters 13 and 16 if the parental groups (1 or 2) are the same, and *connections*$(13, 16,$FALSE$)$ represents the connections between the same subclusters where the parental assignments are not the same. M is a set of IBD segments indexed by database individual.

```
procedure SUBCLUSTER_CONNECTIONS(M)
    Let connections(a, b, p) represent a connection between subclusters a and b
        and p is TRUE if and only if the subcluster parent assignments are the same
    for m ∈ M do // for each database individual m
        // there are T (1 or more) segments shared between m and the proband
        Let S = [s_1, s_2, ..., s_T] be the T segments shared between m and the proband
        for i ∈ 1, 2, ..., T do
            for j ∈ i + 1, i + 2, ..., T do
                // increase connection between two subcluster parent groups
                parent_aligned ← s_i.subcluster.parent = s_j.subcluster.parent
                connections(s_i.subcluster, s_j.subcluster, parent_aligned) += m.cM
    return connections
```

Algorithm 11 Join N subclusters into connected components. B is a set of N subclusters. *connections* is a matrix that defines whether any pair of subclusters in B are connected (by a pair of IBD segments in either subcluster that are shared between the proband and the same database individual).

```
procedure CONNECTED_COMPONENTS(connections)

    N ← number of subclusters in connections

    // Let C initially be a list of singleton lists
    // Each C_i will be a list of subclusters in a connected component
    C ← [[1], [2], [3], ..., [N]] // this is [C_1, C_2, ..., C_N]

    for i ∈ 1, 2, ..., |C| do
        for j ∈ 1, 2, ..., i − 1 do
            for B_i ∈ C_i do // B_i is one of the subclusters in component C_i
                for B_j ∈ C_j do // B_j is one of the subclusters in component C_j
                    if connections(B_i, B_j, TRUE) + connections(B_i, B_j, FALSE) ≠ 0 then
                        // C_i and C_j are connected through B_i and B_j
                        C_i ← C_i ∪ C_j
                        C_j ← ∅
                        (we can exit the inner two loops)

    return all subcluster sets in C that are not empty
```

Algorithm 12 Phase the proband's entire genome based on the parent assignment (parent 1 or 2) to each segment, which was assigned in CREATE_SUPERCLUSTERS. if there are enough (weighted) overlapping IBD segments to infer the phase and/or genotype calls from the alleles of overlapping IBD segments, override the original call (or the imputed genotype in the pre-phased data when the proband's genotype is missing). In our experiments, we use a weighted sum of 1.0 as the evidence threshold.

```
procedure PHASE_PROBAND(P,M)

    for each SNP d do
        Let A_1 ← set of IBD segments s ∈ M that include d and assigned to parent 1
        Let a_1 ← majority allele among A_1
        Let e_1 ← ∑_{s∈A_1} WEIGHT(s,d) // measure of IBD evidence supporting allele 1
        Let A_2 ← set of IBD segments s ∈ M that include d and assigned to parent 2
        Let a_2 ← majority allele among A_2
        Let e_2 ← ∑_{s∈A_2} WEIGHT(s,d) // measure of IBD evidence supporting allele 2
        if e_1 ≥ EVIDENCE_REQ and e_2 ≥ EVIDENCE_REQ then // we use 1.0
            // Ignore the original genotype
            assign the phased genotype (a_1, a_2) to the proband P at d
        else if e_1 ≥ EVIDENCE_REQ then
            if the pre-phased genotype for d in P is homozygous opposite of a_1 then
                assign phased het to P, a_1 for parent side 1 // Correct hom → het call
            else
                retain the pre-phased genotype for d, assign a_1 to parent 1
        else if e_2 ≥ EVIDENCE_REQ then
            (as above, for parent 2 instead of parent 1)
        else
            retain the same genotype as the pre-phased data in P.
                If it is heterozygous, let the side with more IBD
                (compare e_1 to e_2) have its preferred allele.
                If e_1 = e_2 (a common case is they're both 0), then
                retain the pre-phased relationship between site d and
                the previous heterozygous site in the proband.
```
